# Proteomic analysis of fatty liver induced by starvation of medaka fish larvae

**DOI:** 10.1247/csf.23014

**Published:** 2023-06-27

**Authors:** Tomoyo Ikeda, Tokiro Ishikawa, Satoshi Ninagawa, Tetsuya Okada, Masaya Ono, Kazutoshi Mori

**Affiliations:** 1 Department of Biophysics, Graduate School of Science, Kyoto University, Kyoto 606-8502, Japan; 2 National Cancer Center Research Institute, Tokyo 104-0045, Japan

**Keywords:** amino acid catabolism, β-oxidation, triacylglycerol, cholesterol, export

## Abstract

When medaka fish (*Oryzias latipes*) larvae are grown in the absence of exogenous nutrition, the liver becomes dark and positive to Oil Red O staining from 7 days post-hatch (dph). We determined the mechanism of this starvation-induced development of fatty liver by proteomic analysis using livers obtained from larvae grown in the presence or absence of 2% glucose at 5 dph. Results showed that changes in the expression levels of enzymes involved in glycolysis or the tricarboxylic acid cycle were modest, whereas the expression levels of enzymes involved in amino acid catabolism or β-oxidation of fatty acids were significantly elevated, suggesting that they become major energy sources under starvation conditions. Expression levels of enzymes for the uptake and β-oxidation of fatty acids as well as synthesis of triacylglycerol were elevated, whereas those for the synthesis of cholesterol as well as export of cholesterol and triacylglycerol were decreased under starvation conditions, which explains the accumulation of triacylglycerol in the liver. Our results provide the basis for future research to understand how gene malfunction(s) affects the development of fatty liver, which can lead to nonalcoholic steatohepatitis and then to liver cirrhosis.

## Introduction

We use medaka fish (*Oryzias latipes*) as a vertebrate model organism in the analysis of the unfolded protein response (UPR), a series of translational and transcriptional programs that couple with intracellular signaling from the endoplasmic reticulum (ER) to the nucleus to maintain the homeostasis of the ER, where secretory and transmembrane proteins destined for the secretory pathway are folded and assembled (Ishikawa *et al.*, 2011). The UPR is triggered when three types of transmembrane protein in the ER, namely IRE1, PERK and ATF6, sense ER stress, which is characterized by the accumulation of unfolded and misfolded proteins in the ER (Mori, 2009). IRE1 consists of IRE1α and IRE1β, and ATF6 consists of ATF6α and ATF6β in both medaka and mammals. Major transcriptional targets of the UPR are ER-localized molecular chaperones and folding enzymes (collectively termed ER chaperones hereafter). Transcriptional induction of ER chaperones is mediated by the IRE1 arm in yeast, worm and fly, but by the ATF6 arm in medaka and mice (Mori, 2022).

Newly hatched fish are usually grown in a fish tank without water flow using egg water (0.3% NaCl, 0.02% NaHCO_3_) with powdered bait for 7–10 days prior to transfer into a water-recirculating system. We noticed that the livers of hatched fish kept growing in egg water without powdered bait—namely under starvation conditions—became dark. To our knowledge, this starvation-induced development of fatty liver in medaka fish has not been previously reported. Here, we investigated the mechanism underlying this color change.

## Materials and Methods

### Fish

Medaka southern strain Cab was used as WT fish. Fish were maintained in a recirculating system with 14:10 h light/dark cycle at 27.5°C. All experiments were performed in accordance with the guidelines and regulations established by the Animal Research Committee of Kyoto University (approval number: H2819).

Hatched medaka larvae were grown in a 100-mm dish with a complete (100%) daily change of egg water (0.3% NaCl, 0.02% NaHCO_3_) containing or not containing 2% glucose at 28°C. At 0, 4, 7 and 9 days post-hatch (dph), medaka larvae were fixed in 4% paraformaldehyde in PBS overnight at 4°C, washed with 1 × PBS, rinsed with 60% isopropanol for 1 min and then stained with 1.8 mg/ml Oil Red O in 60% isopropanol for 25 min. The samples were washed twice with 60% isopropanol for 15 min each, and then with 1 × PBS for 10 min. Images were obtained using a fluorescence stereomicroscope (M205FA; Leica, Wetzlar, Germany) with a camera (Leica DFX310FX) and acquisition software (Leica LAS AF).

### Mass spectrometric analysis

Dissected medaka livers were fixed with 100% methanol, dried in a Savant SpeedVac, and dissolved in 400 μl of 1% sodium deoxycholate, 1 M urea, 50 mM NH_4_HCO_3_ (Wako, 018-21742) and 2 μg of Sequencing Grade Modified Trypsin (Promega, V5113). After 20-h digestion at 37°C, 80 μl of 5% formic acid was added, followed by centrifugation at 15,000 g for 10 min at 4°C. The supernatants were collected, incubated with 480 μl of ethyl acetate (Wako, 055-03115), and centrifuged at 15,000 g for 10 min at 4°C. 400 μl of the water layer containing digested peptides were collected, dried in a Savant SpeedVac, and dissolved in 50 μl of 0.1% formic acid. 10 μg of digested peptides in 20 μl of 0.1% formic acid were applied to C18 tips (Nikkyo Technos, NTCR-KT200-C18-2). After washing 2 times with 0.1% formic acid containing 2% acetonitrile (Wako, 016-19854), digested peptides were eluted from C18 tips with 0.1% formic acid containing 80% acetonitrile, and dried. These steps were performed using low absorption tips (Gilson, F171301) and tubes (Sumitomo Bakelite, MS-4215M). Two-dimensional image-converted analysis of liquid chromatography and mass spectrometry (2DICAL) shotgun proteomics analysis was performed as described previously (Ono *et al.*, 2012).

### Data Availability

The data of our proteomic analysis have been deposited in jPOSTdb (https://globe.jpostdb.org/) with ID = JPST002120 (PXD041411).

### Statistics

Statistical analysis was conducted using Student’s t-test for relative expression levels determined from the values of identified peptides in starved and non-starved livers. *p<0.05, **p<0.01 and ***p<0.001 for all figures.

## Results

**Fig. 1:**
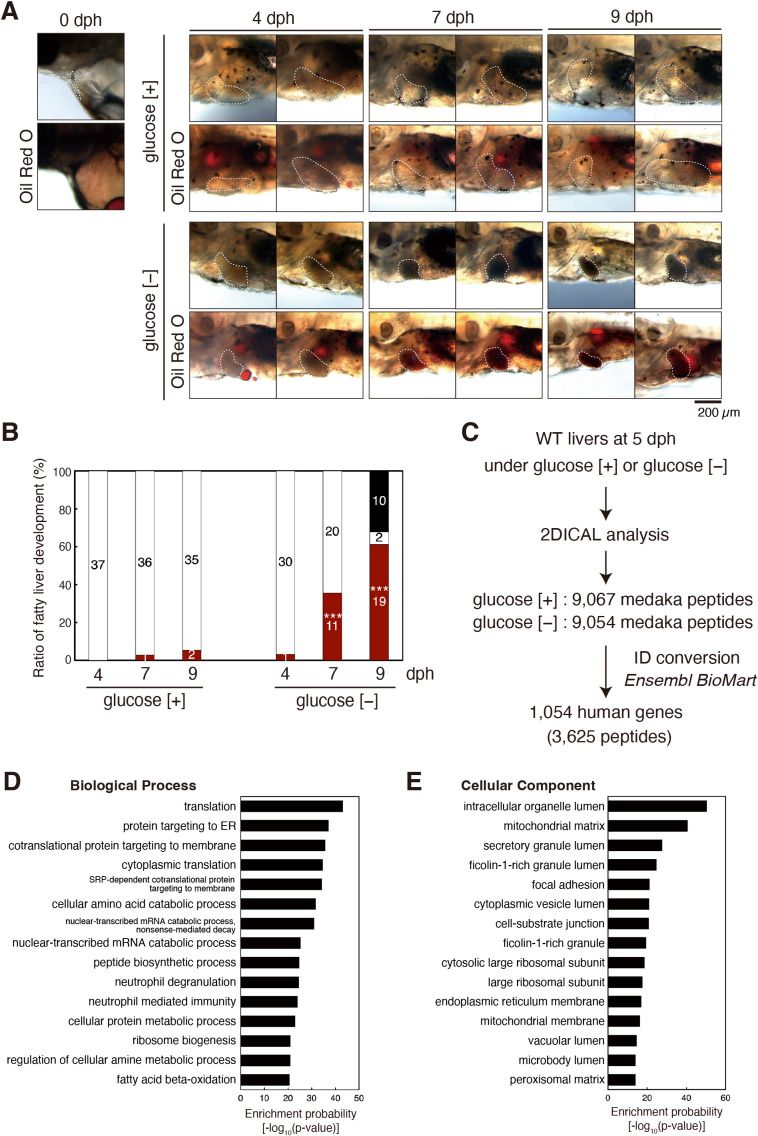
Development of fatty liver upon starvation (A) A part of medaka larvae grown in egg water containing [+] or not containing [–] 2% glucose was photographed at 0, 4, 7 and 9 dph before or after Oil Red O staining. The livers are surrounded by white broken lines. Scale bar: 200 μm. (B) 37 and 31 medaka larvae were grown in egg water containing [+] and not containing [–] 2% glucose, respectively. The number of medaka larvae carrying fatty liver (i.e. dark liver) was counted and is shown as a ratio (%). Red, white, and black bars indicate larvae carrying fatty liver, larvae carrying normal liver and dead larvae, respectively, with the actual number of larvae. (C) Strategy for the proteomic analysis of fatty liver is shown. (D) Top 15 enriched biological processes resulting from GO term enrichment analysis of 1,054 human genes in (C) are shown. (E) Top 15 enriched cellular components resulting from GO term enrichment analysis of 1,054 human genes in (C) are shown.

To avoid individual differences in food intake, we used 2% glucose instead of powdered bait for rearing. When medaka larvae were maintained in egg water containing 2% glucose, they grew normally similarly to the case when reared with powdered bait. In contrast, when medaka larvae were grown in egg water not containing 2% glucose, the liver started to become dark from 5 dph (Fig. 1A and 1B). After staining with Oil Red O, the dark liver became dark red, suggesting that the dark liver indicates fatty liver and that this phenomenon represents starvation-induced steatosis (Fig. 1A and 1B). In this connection, we previously showed that injection of tunicamycin, which induces ER stress by inhibiting protein *N*-glycosylation (Kaufman, 1999), into ATF6α-knockout mice produced Oil Red O-staining-positive livers, resulting from steatosis and lipid droplet formation (Yamamoto *et al.*, 2010). Fatty livers were hardly observed in larvae grown in the presence of 2% glucose, whereas 3%, 35%, and 61% of larvae exhibited fatty livers at 4, 7, and 9 dph, respectively, when grown in the absence of glucose (Fig. 1A and 1B).

To gain insight into the underlying mechanism, we isolated livers from 173 and 155 larvae grown in the presence and absence of 2% glucose, respectively, at 5 dph (namely at the starting time point of steatosis). Proteins were extracted from mixed 173 and 155 livers, denatured, and digested with trypsin, by which we obtained 48.1 μg and 47.0 μg of peptides, respectively. Ten micrograms each of digested peptides were subjected to shotgun proteomics analysis, termed 2DICAL (Ono *et al.*, 2012), which was conducted once. Liquid Chromatography/Mass Spectrometry analysis produced data of mass to charge ratio (*m/z*), retention time, and peak intensity for each digested peptide together with its medaka protein ID based on the Ensembl genome database (http://www.ensembl.org). These three data from one sample (namely 9,067 peptides derived from 173 livers of larvae grown in the presence of 2% glucose) were converted to a two-dimensional image with *m/z* and retention time as X and Y axis, respectively, and with peak intensity as shade. Similarly, these three data of a different sample (9,054 peptides derived from 155 livers of larvae grown in the absence of 2% glucose) were also converted to a two-dimensional image. Subsequently, algorithms were applied to ensure the reproducibility of data of *m/z* and retention time between different samples. As a result, we successfully determined the precise difference in peak intensity of each spot (derived from the same peptide) between two samples, and then expressed the difference as a relative expression level. 2DICAL analyzes large numbers of digested peptides simultaneously without using isotopic labeling. Note that the number of obtained peptides varies from protein to protein and that each relative expression level is presented as a dot in a boxplot.

**Fig. 2:**
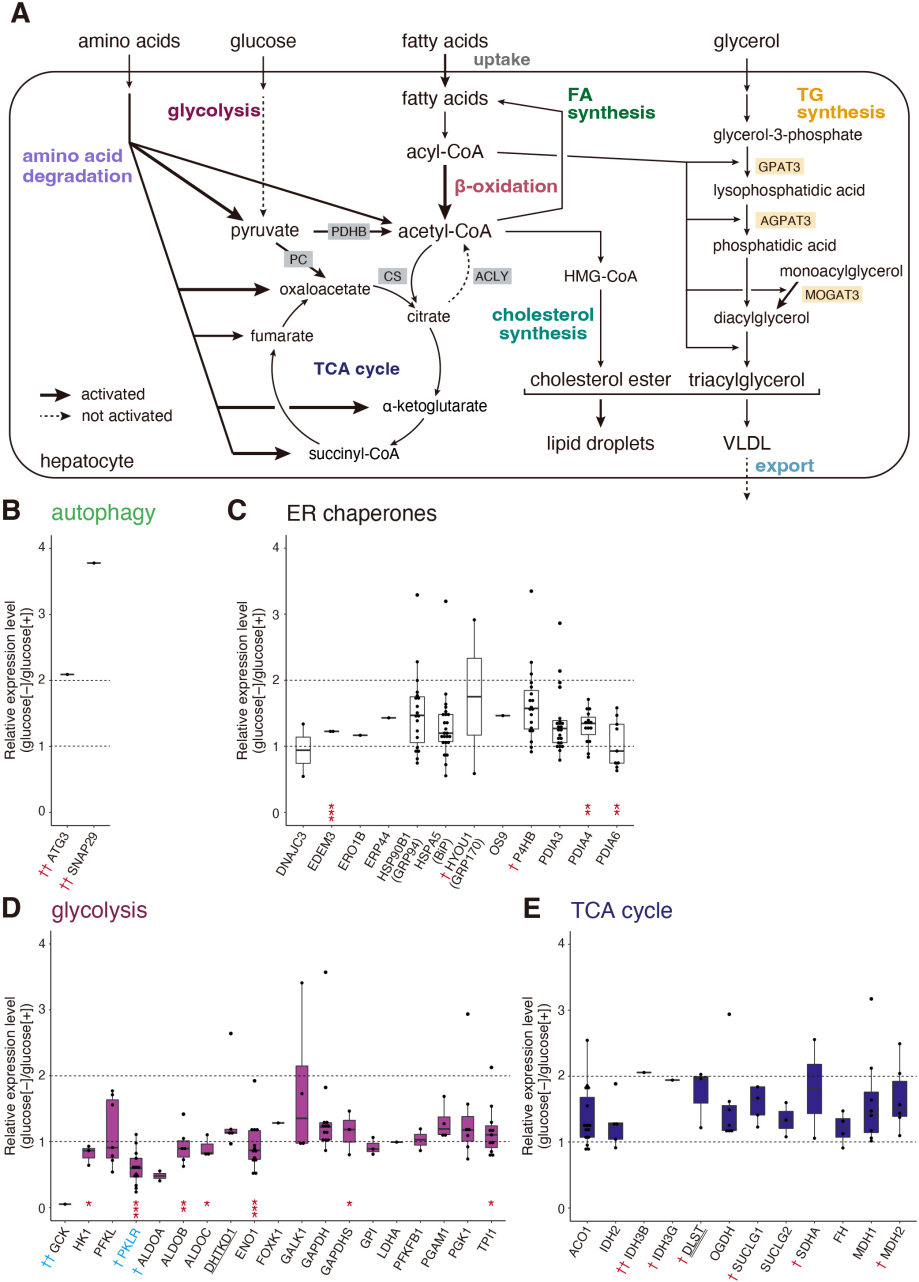
Effect of starvation on levels of classical autophagy gene products, ER chaperones, enzymes involved in glycolysis, and enzymes involved in the TCA cycle in the liver (A) Metabolisms in the liver are schematically presented. (B)–(E) Expression levels of classical autophagy gene products (B), various ER chaperones (C), various enzymes involved in glycolysis (D), and various enzymes involved in the TCA cycle (E) in starved liver are shown relative to those in non-starved liver by Dot-boxplots. Red † and †† denote enzymes whose expression levels were increased more than 1.5-fold and 2-fold, respectively, upon starvation, whereas blue † and †† denote enzymes whose expression levels were decreased more than 1.5-fold and 2-fold, respectively, upon starvation. The blue color of the enzyme PKLR indicates a decrease with significance. The underlining of enzyme DHTKD1 and double-underlining of enzyme DLST denote that they are also involved in amino acid catabolism.

We analyzed only their human orthologues because of the much greater volume of information in the literature. 9,067 and 9,054 peptides were assigned to 3,625 human peptides corresponding to 1,054 human genes using Ensembl BioMart (Ensembl Genes 97 version) (https://asia.ensembl.org/info/data/biomart/index.html) (Fig. 1C). Gene ontology (GO) term enrichment analysis of these genes was conducted using Enrichr (2021 version) (Xie *et al.*, 2021), and GO terms were obtained using Metascape (version 3.5.20230101) (Zhou *et al.*, 2019). The resulting top 15 enriched ‘Biological Process’ and ‘Cellular Component’ are shown for reference (Fig. 1D and 1E). Of note, the expression levels of classical autophagy gene products tended to be increased, as expected (Fig. 2B), whereas those of 12 ER chaperones belonging to GO: 0034975 (protein folding in endoplasmic reticulum), which are targets of the UPR (Mori, 2009), were not altered after starvation, suggesting little induction of ER stress under this condition (Fig. 2C).

The liver is considered to produce energy via catabolism of glycogen (glycolysis), amino acids, and fatty acids (Fig. 2A) (Cahill, 2006; Owen *et al.*, 1969). GO: 0006096 (glycolytic process) includes 19 genes. Among these, 4 enzymes, namely glucokinase (GCK), hexokinase-1 (HK1), 6-phosphofructokinase-liver type (PFKL), and pyruvate kinase L/R (PKLR), participate in glycolysis but not in gluconeogenesis, and PKLR produces pyruvate and ATP from phosphoenolpyruvate. As their levels were rather decreased (Fig. 2D), glycolytic production of ATP appeared not to be activated under starvation conditions (denoted by a broken arrow underneath glucose in Fig. 2A). GO: 0006099 [tricarboxylic acid (TCA) cycle] includes 12 genes, and produces NADH for the electron transport chain. The levels of these enzymes were not significantly altered upon starvation (Fig. 2E).

**Fig. 3:**
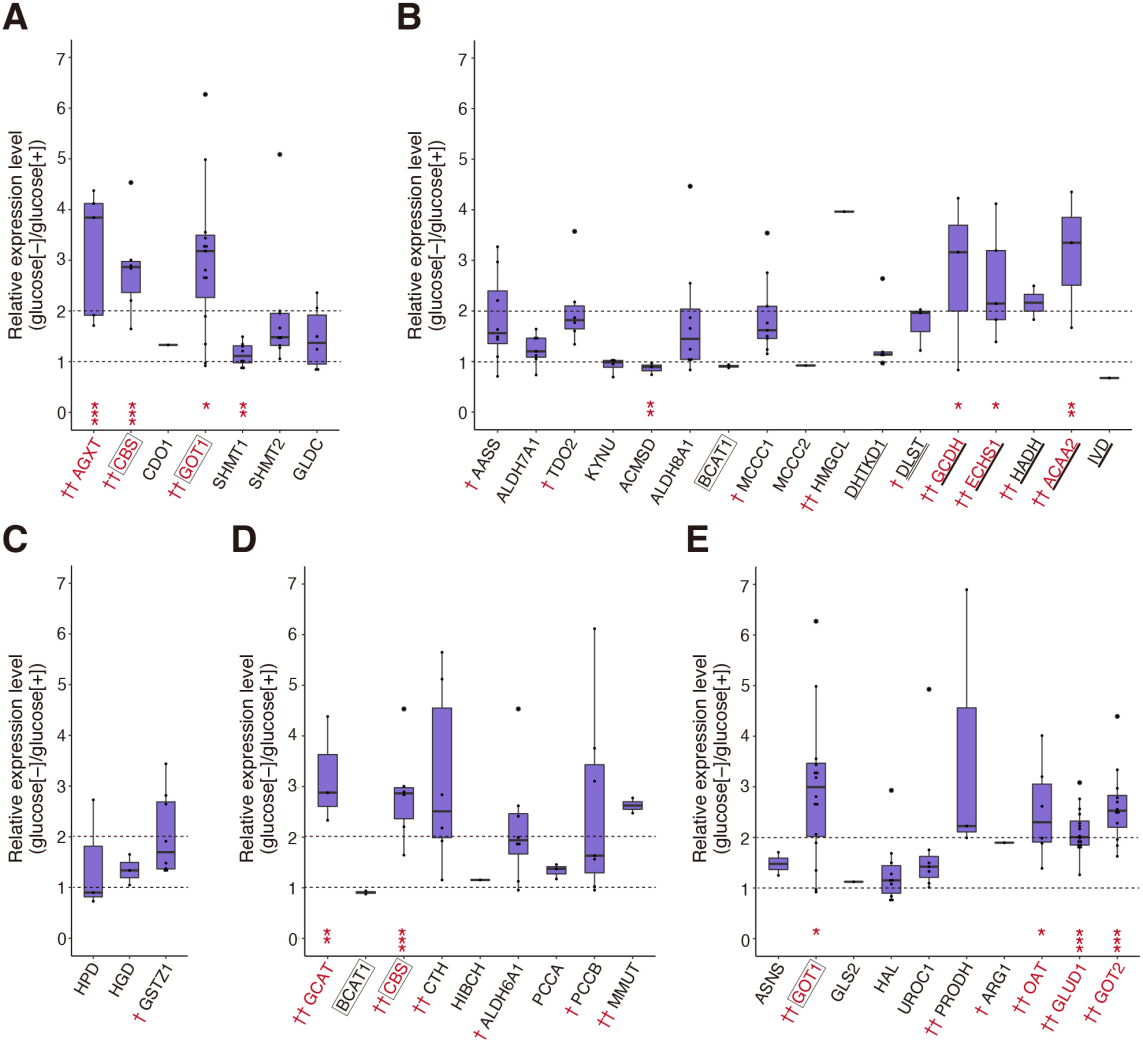
Effect of starvation on levels of catabolic enzymes of amino acids (A)–(E) Expression levels of various enzymes producing pyruvate (A), acetyl-CoA (B), acetyl-CoA and fumarate (C), succinyl-CoA or succinyl-CoA plus acetyl-CoA (D), and oxaloacetate or α-ketoglutarate (E) in starved liver are shown relative to those in non-starved liver by Dot-boxplots. Red † and †† denote enzymes whose expression levels were increased more than 1.5-fold and 2-fold, respectively, upon starvation. Red coloring of the enzyme names indicates a significant increase. Three enzymes (CBS, GOT1, and BCAT1) operating in two categories are boxed with black color. Enzymes underlined, double-underlined, and thick-underlined also participate in glycolysis, the TCA cycle, and β-oxidation, respectively.

**Fig. 4:**
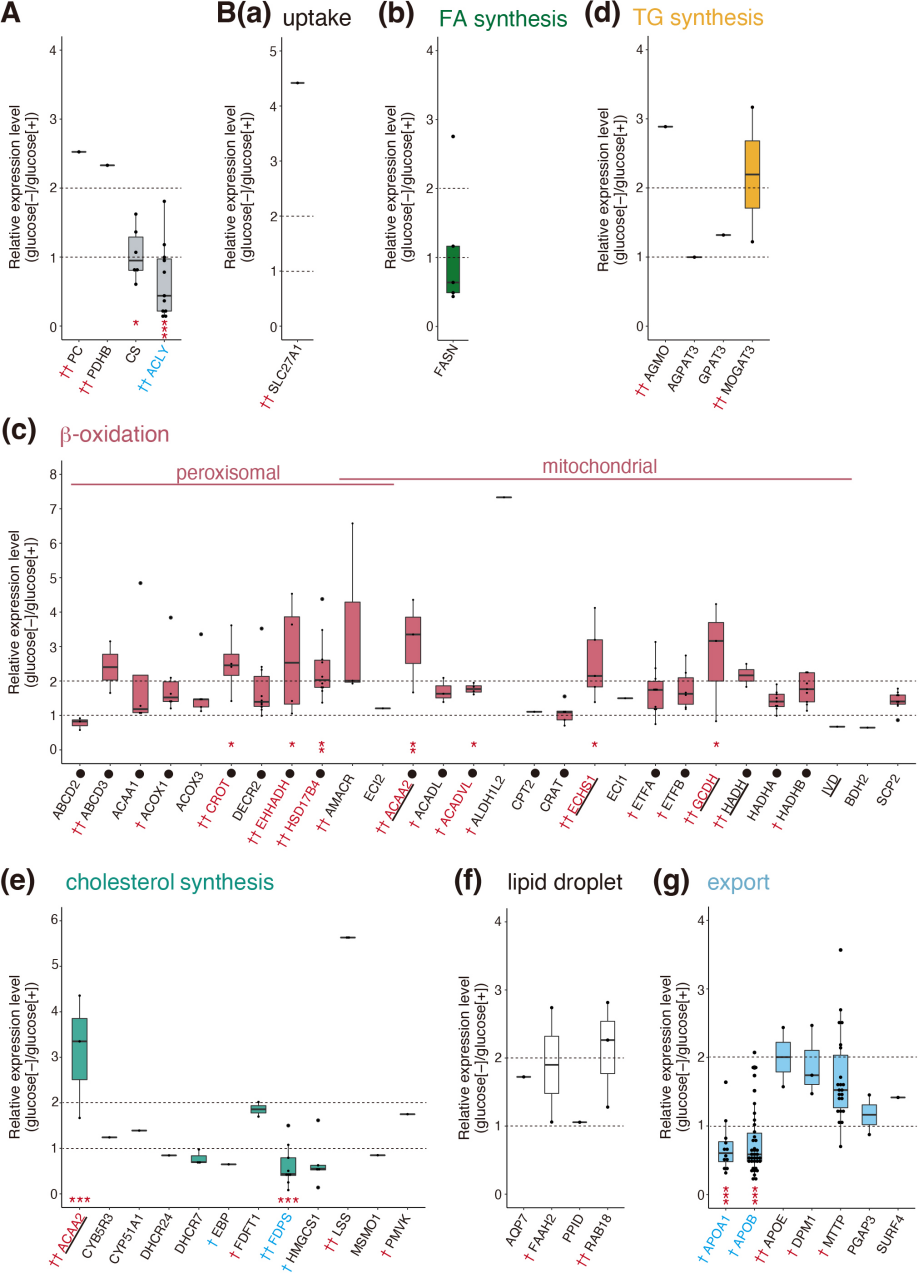
Effect of starvation on levels of enzymes involved in metabolism of fatty acids, triacylglycerol, and cholesterol (A) (B) Expression levels of enzymes connecting glycolysis, the TCA cycle and acetyl-CoA (A) as well as those involved in metabolism of fatty acids, triacylglycerol, and cholesterol (B) in starved liver are shown relative to those in non-starved liver by Dot-boxplots. Red † and †† denote enzymes whose expression levels were increased more than 1.5-fold and 2-fold, respectively, upon starvation, whereas blue † and †† denote enzymes whose expression levels were decreased more than 1.5-fold and 2-fold, respectively, upon starvation. Red and blue coloring of the enzyme names indicate an increase and decrease, respectively, with significance. Black closed circles denote enzymes regulated by PPARα. Thick-underlined enzymes also participate in amino acid catabolism.

Table I summarizes the catabolic enzymes of 20 amino acids, categorized according to their final products (A–E) based on literature search. Purple-colored names indicate enzymes whose peptides were identified by the current proteomic analysis. Underlined, double-underlined, and thick-underlined names indicate enzymes also involved in glycolysis, the TCA cycle, and β-oxidation, respectively. Levels of catabolic enzymes producing pyruvate (Table IA and Fig. 3A), those producing succinyl-CoA or succinyl-CoA plus acetyl-CoA (Table ID and Fig. 3D), and those producing oxaloacetate or α-ketoglutarate (Table IE and Fig. 3E) were significantly elevated under starvation conditions (denoted by thick arrows in Fig. 2A), whereas those producing acetyl-CoA (Table IB and Fig. 3B) and those producing acetyl-CoA and fumarate (Table IC and Fig. 3C) tended to be elevated (denoted by intermediate arrows in Fig. 2A), suggesting that amino acid catabolism is generally activated upon starvation. Although only one peptide each was identified for pyruvate carboxylase (PC) converting pyruvate to oxaloacetate and for pyruvate dehydrogenase (PDHB) converting pyruvate to acetyl-CoA (Fig. 2A), their levels were increased more than 2-fold (Fig. 4A). The level of citrate synthase (CS) converting acetyl-CoA to citrate was not altered, whereas that of ATP citrate lyase (ACLY) converting citrate to acetyl-CoA was significantly decreased (Fig. 2A and 4A).

Fatty acid (FA) levels in the liver are regulated by a balance between uptake, synthesis, catabolism (β-oxidation), and export (Fig. 2A) (Bradbury, 2006; Duval *et al.*, 2007; Reddy and Rao, 2006). Large amounts of FA are released from the adipose tissue under starvation conditions (Kersten *et al.*, 1999) and FA uptake is mediated by members of the SLC27A family, namely A1, A2, A3, A4, A5, and A6 as well as fatty acid translocase (CD36). Although only one peptide of SLC27A1 was identified, its expression level was increased more than 4-fold [Fig. 4B(a)]. In contrast, the level of fatty acid synthase (FASN), the rate limiting enzyme in the synthesis of FA (Alves-Bezerra and Cohen, 2017), tended to be decreased upon starvation [Fig. 4B(b)]. In marked contrast, the levels of enzymes responsible for β-oxidation [total of 28 genes included in GO: 0006635 (fatty acid β-oxidation)] were generally increased [Fig. 4B(c)]. As the enzymes marked with closed circles in Fig. 4B(c) are known to be targets of PPARα (Rakhshandehroo *et al.*, 2010), PPARα-mediated induction of β-oxidation appears to be activated upon starvation (denoted by the thick arrow underneath acyl-CoA in Fig. 2A).

Triacylglycerol (TG) is synthesized mainly in the liver via three cycles of acylation of glycerol-3-phosphate using acyl-CoA, which is produced from fatty acids (Fig. 2A). The levels of enzymes involved in TG synthesis (Shi and Cheng, 2009) tended to be increased upon starvation [Fig. 4B(d)]. GO:0006695 (cholesterol biosynthetic process) includes enzymes involved in cholesterol synthesis. The level of acetyl-CoA acyltransferase (ACAA2) involved in both β-oxidation and cholesterol synthesis (Kiema *et al.*, 2014) was increased more than 3-fold, whereas that of farnesyl diphosphate synthase (FDPS) was decreased significantly [Fig. 4B(e)].

The levels of enzymes involved in lipid droplet formation tended to be increased upon starvation [Fig. 4B(f)], whereas those of apolipoprotein A1 (APOA1) and apolipoprotein B (APOB), key proteins for the export of TG and cholesterol ester-containing high density lipoprotein (HDL) and very low density lipoprotein (VLDL), respectively, were decreased significantly [Fig. 4B(g)].

## Discussion

In this study, we found that the rearing of medaka larvae under starvation conditions induced the development of fatty liver. The liver is considered to produce energy via the catabolism of glycogen (glycolysis), amino acids, and fatty acids. Our proteomic analysis showed that changes in the expression levels of enzymes involved in glycolysis (Fig. 2D) and the TCA cycle (Fig. 2E) were modest, whereas the expression levels of many enzymes involved in amino acid catabolism (Fig. 3) and β-oxidation of FA [Fig. 4B(c)] were elevated significantly upon starvation. Of note, many upregulated enzymes involved in β-oxidation are known to be regulated by PPARα, which mediates the adaptive response to fasting (Kersten *et al.*, 1999; Pawlak *et al.*, 2015). In contrast, FA synthesis appeared to be down regulated upon starvation [Fig. 4B(b)]. These results suggest that amino acid catabolism and β-oxidation give rise to major sources of energy production under starvation conditions.

TG is accumulated in fatty liver. We found that the expression levels of enzymes involved in TG synthesis tended to be elevated [Fig. 4B(d)]. In contrast, although peptides of HMG-CoA synthase and HMG-CoA reductase, rate-limiting enzymes for cholesterol synthesis, were not detected by our current proteomic analysis, the activity of HMG-CoA reductase was previously shown to be decreased under fasting conditions in rat livers (Kelley and Story, 1985). In addition, the expression level of farnesyl diphosphate synthase (FDPS), whose product (farnesyl pyrophosphate) is a key intermediate in cholesterol and sterol biosynthesis, was significantly decreased [Fig. 4B(e)], suggesting that cholesterol synthesis level is lowered upon starvation. An imbalance between increased TG synthesis and decreased cholesterol synthesis may suggest a decreased synthesis of HDL and VLDL containing TG and cholesterol, leading to the accumulation of free TG in the liver. Furthermore, expression levels of APOA1 and APOB, major components of HDL and VLDL in the export of TG and cholesterol, were significantly decreased [Fig. 4B(g)]. It was shown previously that the knockdown of APOB resulted in the accumulation of TG in mouse liver (Koornneef *et al.*, 2011; Tadin-Strapps *et al.*, 2011). It was also shown that liver fat content was inversely related to the rate of APOB-100 synthesis in severely malnourished children (Badaloo *et al.*, 2005). These results explain the TG accumulation-mediated development of fatty liver under starvation conditions.

Recently, deprivation of exogenous nutrients (powdered bait AP100) from 5 days post-fertilization (dpf) was shown to cause the development of fatty liver from 7 dpf in zebrafish (*Danio rerio*) larvae (Xu *et al.*, 2021). RNA-seq analysis showed that, compared with normal liver, fatty liver has (1) increased levels of mRNA encoding FA uptake, (2) increased levels of mRNA encoding lipogenesis, namely pparab mRNA encoding the zebrafish orthologue of human PPARα, (3) decreased levels of mRNA encoding FA metabolism, mostly β-oxidation, and (4) decreased levels of mRNA encoding lipid transport. Results (1) and (4) are consistent with those of our proteomic analysis, shown in Fig. 4B(a) and 4B(g), respectively. Although peptides of PPARα were not detected by our proteomic analysis, the increase in levels of PPARα targets [Fig. 4B(c)] is consistent with result (2).

Of note, result (3) contradicts our results shown in Fig. 4B(c), which shows an increase in the levels of enzymes involved in β-oxidation. This might be due to the difference in sampling timing—Xu *et al.* used fatty livers, whereas we used livers that were starting to accumulate TG (5 dph, Fig. 1A–C)—or to the difference in a diet for control larvae, namely protein-containing powdered bait versus 2% glucose. In this connection, we found a variation in how β-oxidation is associated with fatty liver in the literature: some reports showed elevation of β-oxidation in patients with nonalcoholic steatohepatitis (Bugianesi *et al.*, 2005; Dasarathy *et al.*, 2011; Sanyal *et al.*, 2001), whereas others showed no change (Kotronen *et al.*, 2009) or even a decline (Croci *et al.*, 2013; Moore *et al.*, 2022) in β-oxidation in those patients.

Our analysis started from the observation that the liver of medaka larvae became dark when they were grown without powdered bait. This darkness may be associated with bile acid, which is synthesized and secreted by hepatocytes, given previous findings that the liver became dark through the accumulation of bile acid when bile acid transporters were down-regulated in zebrafish larvae (Wang *et al.*, 2022). Nonetheless, we found that the levels of 5 bile acid transporters (ABCB11, ABCB4, ABCC11, ABCC4, ABCD3) were not decreased in the liver of larvae grown in the absence of glucose (data not shown). In addition, the gall bladder, which stores bile acid, became bigger and is considered to contain more bile acid in larvae grown in the absence of glucose but its dark green color was not changed. The cause of the darkness remains to be determined.

We showed in this report that the dark liver represents the fatty liver and determined the mechanism of starvation-induced development of the fatty liver. Our results provide a basis for future research into the effects of gene malfunction(s) on liver steatosis, which can lead to the development of nonalcoholic steatohepatitis and then to the development of liver cirrhosis. The threat of these changes in human health is increasing.

## Figures and Tables

**Table I:**
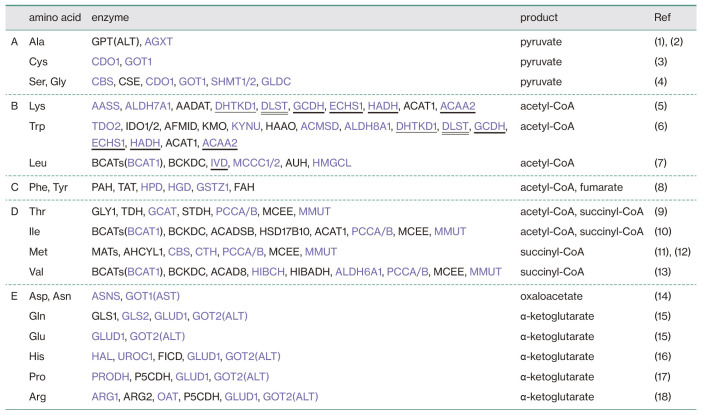
List of catabolic enzymes of 20 amino acids Various catabolic enzymes are classified into 5 categories (A)–(E) based on final products. Purple coloring of the enzyme names indicate that their peptides were identified by proteomic analysis. Enzymes underlined, double-underlined, and thick-underlined also participate in glycolysis, the TCA cycle, and β-oxidation, respectively. *References* (1) Oppici *et al.*, 2015 (2) Jose *et al.*, 2013 (3) Werge *et al.*, 2021 (4) Narkewicz *et al.*, 1996 (5) Leandro and Houten, 2020 (6) Shibata, 2018 (7) Manoli and Venditti, 2016 (8) Kim *et al.*, 2000 (9) Tang *et al.*, 2021 (10) Murín *et al.*, 2009a (11) Clare *et al.*, 2021 (12) Leal *et al.*, 2004 (13) Murín *et al.*, 2009b (14) Marchese *et al.*, 2018 (15) Yoo *et al.*, 2020 (16) Brosnan and Brosnan, 2020 (17) Du *et al.*, 2021 (18) Andersen *et al.*, 2016

## Abbreviations

2DICAL: two-dimensional image converted analysis of liquid chromatography and mass spectrometry
APOA1: apolipoprotein A1
APOB: apolipoprotein B
dpf: day post-fertilization
dph: day post-hatch
ER: endoplasmic reticulum
FA: fatty acid
GO: gene ontology
HDL: high density lipoprotein
TCA: tricarboxylic acid
TG: triacylglycerol
UPR: unfolded protein response
VLDL: very low density lipoprotein
